# Biomass Fuel Use and Cardiac Function in Nepali Women

**DOI:** 10.5334/gh.405

**Published:** 2020-02-07

**Authors:** Jasleen Tiwana, Catherine Benziger, Laura Hooper, Karl Pope, Vijay Alurkar, Ramchandra Kafle, Tula R. Sijali, John R. Balmes, Joel D. Kaufman, Michael N. Bates

**Affiliations:** 1 Division of Cardiology, University of Washington, Seattle, WA, US; 2 St. Mary’s Heart and Vascular Center, Essentia Health, Duluth, MN, US; 3 Department of Medicine, Swedish Hospital, Seattle, WA, US; 4 Division of Environmental Health Sciences, School of Public Health, University of California, Berkeley, US; 5 Department of Medicine, Manipal College of Medical Sciences and Manipal Teaching Hospital, Pokhara, NP; 6 Institute for Social and Environmental Research-Nepal (ISER-N), Pokhara, NP; 7 Department of Medicine, University of California, San Francisco, CA, US; 8 Departments of Environmental and Occupational Health Sciences, Medicine, and Epidemiology, University of Washington, Seattle, WA, US

**Keywords:** Household air pollution, echocardiography, cardiovascular changes

## Abstract

**Background:**

Exposure to household air pollution (HAP) from cooking with biomass fuel affects billions of people. We hypothesized that HAP from woodsmoke, compared to other household fuels, was associated with adverse cardiovascular outcomes, of which there have been few studies.

**Methods:**

A cross-sectional study was completed in 299 females aged 40–70 years in Kaski District, Nepal, during 2017–18. All participants underwent a standard 12-lead ECG, ankle and brachial systolic blood pressure measurement, and 2D color and Doppler echocardiography. Current stove type was confirmed by inspection. Blood pressure, height, and weight were measured using a standardized protocol. Hypertension was defined as ≥140/90 mmHg or prior diagnosis. Hemoglobin A1c (HbA1c) was obtained, with diabetes mellitus defined as a prior diagnosis or HbA1C ≥ 6.5%. We used adjusted linear and logistic multivariable regressions to examine the relationship of stove type with cardiac structure and function.

**Results:**

The majority of women primarily used liquified petroleum gas (LPG) stoves (65%), while 12% used biogas, and 23% used wood-burning cook-stoves. Prevalence of major cardiovascular risk factors was 35% with hypertension, 19% with diabetes mellitus, and 15% current smokers. After adjustment, compared to LPG, wood stove use was associated with increased indexed left atrial volume (β = 3.15, 95% CI 1.22 to 5.09) and increased indexed left ventricular end diastolic volume (β = 7.97, 95% CI 3.11 to 12.83). There was no association between stove type and systemic hypertension, left ventricular mass, systolic dysfunction, diastolic dysfunction, pulmonary hypertension, abnormal ankle-brachial index, or clinically significant ECG abnormalities.

**Conclusion:**

Biomass fuel use was associated with increased indexed left atrial volume and increased indexed left ventricular diastolic volume in Nepali women, suggesting subclinical adverse cardiac remodeling from HAP in this cross-sectional study. We did not find evidence of an association with hypertension or typical cardiac sequelae of hypertension. Future studies to confirm these results are needed.

## Introduction

Environmental pollution is a significant risk factor for death and disability worldwide. Observational epidemiologic studies have linked ambient air pollution to cardiovascular morbidity and mortality [15,23,29]. Air pollution contributes to systemic oxidative stress and inflammation, lipid oxidation, and metabolism, eventually leading to endothelial dysfunction resulting in accelerated atherosclerosis and propensity for thrombosis [12,25]. In 2016, the World Health Organization (WHO) attributed approximately 4.2 million premature deaths a year to air pollutants, specifically fine particulate matter <2.5 μm in diameter (PM_2.5_) [32]. In developing regions, combustion of biomass (e.g., wood, animal dung, or crop waste), coal, or kerosene for cooking and heating are significant sources of household air pollution (HAP), with an estimated 3 billion people affected worldwide [33]. Prior studies have found associations between household air pollution and hypertension, acute coronary syndrome, non-specific ST depression on electrocardiogram (ECG), and endothelial dysfunction, among other markers for cardiovascular disease [5,11,16,17,18,20,21,31]. Attempts have also been made to detect subclinical changes in cardiac structure and function, with a prior study showing possible increases in left ventricular and left atrial sizes and worse global longitudinal strain [28].

Overall, the link between HAP from the use of solid fuels and cardiovascular disease has not been as thoroughly investigated as outdoor air pollution and cardiovascular disease. Few studies have had the opportunity to take advantage of comprehensive tests that can be offered in a fully equipped hospital setting, with most studies having relied on field evaluations. The objective of our study was to further investigate the relationships between fuels used for cooking and clinical and subclinical cardiovascular changes. The present study, by taking advantage of the infrastructure of and participants in a study of eye diseases taking place in Kaski District, Nepal, had participants undergo an in-hospital suite of cardiovascular tests, including blood pressure measurement, echocardiography, ankle brachial index, and electrocardiogram. Given prior studies of HAP, we expected to find an association between hypertension and cooking with solid fuels, relative to cooking with gas fuels (liquified petroleum gas (LPG) or biogas). As sequelae of hypertension, we hypothesized associations between solid fuel use and increased left ventricular mass and diastolic dysfunction on echocardiography. We also hypothesized that solid fuel use would be associated with higher pulmonary artery systolic pressures as a result of pulmonary disease and diastolic dysfunction. Lastly, we hypothesized an association between lower ankle brachial index (ABI) and solid fuel use.

## Methods

### Study setting and participants

The initial basis for the selection of study participants was households of control participants in a pulmonary tuberculosis (TB) case-control study carried out in and around Kaski District, Nepal [2,4], with the objective of obtaining 300 participants from households, with a balanced distribution of the three main fuels used in this area in primary stoves for cooking: LPG, wood, and biogas. Participants in the present study were required to be women, who do most of the cooking in Nepal, aged 40–70 years.

Once a household was approached and agreed in principle to participate, we obtained demographic details of the female household members. If there was more than one potentially eligible woman in a household, we randomly selected from among them. If the selected woman provided informed consent and was found eligible to participate, she was interviewed at her home using a structured questionnaire and transported to Manipal Hospital, Pokhara city, in a study vehicle. At the hospital, she underwent study procedures in the Department of Cardiology, as well as in the Department of Ophthalmology. The procedures in the latter department are not relevant to this publication and are not covered further here.

If a household did not have an eligible and willing participant, or the participant could not be contacted, then further addresses were approached in the order in which they were listed. No more than one woman per household was recruited. Participants were each paid 400 Nepali rupees (about US$4) for their participation.

### Exclusion criteria

1. There were no exclusions based on race or ethnicity.
2. Breastfeeding women were excluded, because of the use of albuterol for spirometry (not covered in this publication).
3. A number of exclusions were made because they were necessary for the concomitant ocular study:
  - Anyone who was blind, had congenital cataracts, replacement lens surgery, or penetrating eye trauma.
  - Anyone undergoing cancer chemotherapy.
  - Pregnant women were excluded because of the need to dilate pupils for ocular photography.

### Questionnaire

The study questionnaire was administered in person by trained study staff. Data gathered included background demographic information, household use of fuel-based devices (cooking, heating, and lighting), tobacco and alcohol use, education, occupation, markers of wealth and literacy including income, house and land ownership, ability to read and write Nepali, and medical history. The questionnaire was programmed onto laptop computers, using CasicBuilder™ software (West Portal Software Corporation, San Francisco, USA) and extensively field tested. Stove types were confirmed by inspection by study team members.

### Diabetes screening test

Blood samples were obtained at participant homes and were analyzed for hemoglobin A1c (HbA1c) concentration using a MISPA I2 automated analyzer (Agappe Diagnostics, Switzerland). Once the result was available, participants were advised of their result and if the HbA1c reading was ≥ 6.5%, suggestive of diabetes mellitus, were advised to get medical advice. Diabetes status was defined as prior diagnosis of diabetes, as indicated by response to the study questionnaire, or if HbA1c was ≥ 6.5%.

### Blood pressure (BP) measurement

Resting BP measurements were performed using an automated blood pressure cuff (Omron 7 Series BP760N, Kyoto, Japan), according to a standardized protocol. After sitting in a quiet room for at least 5 minutes, participants had their brachial artery BP measured at least three times with a 1-minute interval between measurements. The first blood pressure reading was discarded and the second and third were averaged to calculate systolic BP (SBP) and diastolic BP (DBP). Hypertension status was defined as prior diagnosis of hypertension as indicated on the study questionnaire or if measured BP was ≥ 140/90.

### Electrocardiography

An ECG was performed using electrodes arranged in a standard 12-lead configuration. ECG analysis was performed using the Minnesota Code Classification System to record QT and QTc intervals, conduction abnormalities, arrhythmias, ST segment elevations and depressions, T wave deflections, and presence of Q waves [7,8]. QT interval duration was corrected (QTc) for heart rate using the Bazett formula [10].

### Ankle brachial pressure index (ABI) measurement

Doppler blood pressure was obtained for the bilateral brachial, dorsalis pedis, and posterior tibial arteries (Edan SonoTrax Vascular Doppler 8MHz, Shenzhen, China). ABI was calculated for each leg as the ratio of the highest measurement of ankle SBP (either dorsalis pedis or posterior tibial artery) to the brachial SBP. The presence of peripheral arterial disease was defined as an ABI < 0.9 in one or both legs [13].

### Echocardiography

Ultrasound measurements were performed using a 2D color and Doppler echo system (Siemens ACUSON SC2000 PRIME, Berlin, Germany) for at least three consecutive cardiac cycles. All patients were studied in the left lateral recumbent position after a 10-minute resting period for baseline measurements, according to the recommendations of the American Society of Echocardiography (ASE) [19]. The comprehensive echocardiographic study protocol was designed to assess left ventricular (LV) mass, cardiac chamber sizes, LV ejection fraction, diastolic function, pulmonary artery pressure, and presence of valvular disease. One trained cardiologist performed all the studies to reduce inter-operator variability. Images were re-read in a blinded fashion by cardiologists (JT, CPB) trained in echocardiography.

### Statistical analysis

All data analysis was carried out using Stata (version 14.2, StataCorp LLC, College Station, TX). We began with a general descriptive analysis of all data from the questionnaire and cardiovascular examination. The ranges of values were examined for plausibility and outlying values were either removed or flagged.

Measures of subclinical disease were based on the Multi-Ethnic Study of Atherosclerosis (MESA) protocol [6]. Cardiac structure and function, including left ventricular mass, systolic and diastolic function, were evaluated via echocardiography. We also evaluated valvular disease and pulmonary pressures. Electrocardiography (ECG) was obtained to assess for strain patterns. Ankle brachial pressure index was obtained to evaluate for peripheral vascular disease. BP was measured to evaluate for hypertension.

Linear regression was used to model continuous outcomes (e.g., BP) and logistic regression for binary outcomes (e.g., ST elevation or ST depression). For the primary cookstove analysis, LPG was always used as the reference category. For practical reasons, a common set of covariates, selected *a priori*, was used in each multivariable regression, either linear or logistic regression: age, body mass index (BMI) or body surface area (BSA), education, diabetes, smoking history, urban/rural residence, presence of house heating, and whether fuel was used for lighting, to examine the relationship of stove type with cardiac structure and function. Supplemental linear and logistic regression analyses were performed, including diastolic blood pressure and pulse pressure, in addition to the above-mentioned covariates.

### Ethical oversight

Approvals to conduct this research were obtained from the Institutional Review Boards at the University of California, Berkeley, the University of Washington, Seattle, and the Nepal Health Research Council. No data were obtained from study participants until they had provided informed consent.

## Results

In order to achieve the target of 300 participants, attempts were made to recruit 318 eligible women. Of these, one died, one was too ill to travel to the hospital, and 15 refused, mostly because they said they could not afford the time or the compensation provided was too little. This left 301 participants (participation rate 98%) who underwent study procedures during May 2017 to January 2018. Of these, two were excluded from the analysis because their primary stove type was not LPG, biogas, or wood. The median age of the remaining 299 women was 53 (Interquartile range: 48–60). years. Of these, there were 195 LPG, 35 biogas, and 69 wood stove users (Table 1). In our study population, the overall prevalence of cardiovascular risk factors was 35% with hypertension, 19% with diabetes mellitus, and 15% current smokers. Across the stove use groups, there was no significant difference in age, alcohol use, hypertension, or diabetes. In general, the population that used primarily LPG stoves had higher BMI, had more formal education, were less likely to smoke, lived in urban locations with higher incomes, and were wealthier and more likely to be literate compared to wood stove users. Additional baseline characteristics gathered from the study questionnaire are outlined in Supplemental Table 1.

**Table 1:** Baseline characteristics by primary stove type for 299 Nepali women, Kaski District, Nepal.

	All Stoves (N = 299)	LPG (N = 195)	Biogas (N = 35)	Wood (N = 69)	X^2^ value across stove types

	N (%)	N (%)	N (%)	N (%)	P value

**Age categories (years)**					
40 to <45	35 (12%)	23 (12%)	3 (9%)	9 (13%)	
45 to <50	54 (18%)	38 (19%)	8 (23%)	8 (12%)	
50 to <55	78 (26%)	53 (27%)	10 (29%)	15 (22%)	
55 to <60	55 (18%)	36 (18%)	7 (20%)	12 (17%)	
60 to <65	48 (16%)	33 (17%)	3 (9%)	12 (17%)	
65 to <70	29 (10%)	12 (6%)	4 (11%)	13 (19%)	0.20
**Marital status**					
Married	247 (83%)	162 (84%)	27 (77%)	58 (84%)	
Single	2 (1%)	2 (1%)	0 (0%)	0 (0%)	
Widow	47 (16%)	28 (14%)	8 (23%)	11 (16%)	
Divorced	2 (1%)	2 (1%)	0 (0%)	0 (0%)	0.73
**Monthly income (Nepalese Rupee)**					
<8 K	27 (9%)	10 (5%)	2 (6%)	15 (22%)	
8–16 K	36 (12%)	18 (9%)	4 (11%)	14 (20%)	
16–32 K	90 (30%)	57 (29%)	15 (43%)	17 (25%)	
32–64 K	63 (21%)	47 (24%)	8 (23%)	7 (10%)	
>64 K	33 (11%)	27 (14%)	1 (3%)	5 (7%)	
Refuse/DNK	52 (17%)	36 (18%)	5 (14%)	11 (16%)	0.00
**Living location**					
Urban	177 (59%)	155 (79%)	16 (46%)	6 (9%)	
Rural	122 (41%)	40 (21%)	19 (54%)	63 (91%)	0.00
**Education**					
No formal education	171 (57%)	99 (51%)	17 (49%)	55 (80%)	
Any primary	60 (20%)	38 (20%)	13 (37%)	9 (13%)	
Any secondary	57 (19%)	49 (25%)	4 (11%)	4 (6%)	
Greater than secondary education	10 (3%)	8 (4%)	1 (3%)	1 (1%)	0.00
**Read & write Nepali**	131 (44%)	94 (48%)	21 (60%)	16 (23%)	0.00
**Smoking history**					
Never	205 (69%)	143 (73%)	25 (71%)	37 (54%)	
Current	46 (15%)	24 (12%)	6 (17%)	16 (23%)	
Ex-Smoker	48 (16%)	28 (14%)	4 (11%)	16 (23%)	0.04
**Alcohol history**					
Don’t drink	193 (65%)	118 (61%)	27 (77%)	48 (70%)	
Occasional Drink	71 (24%)	56 (29%)	4 (11%)	11 (16%)	
>1 drink/week for >6 months	35 (12%)	21 (11%)	4 (11%)	10 (14%)	0.08
**BMI categories**					
Underweight (<19 kg/m^2^)	12 (4%)	5 (3%)	0 (0%)	7 (10%)	
Normal (19–25 kg/m^2^)	114 (38%)	63 (32%)	18 (51%)	33 (48%)	
Overweight (25–30 kg/m^2^)	109 (36%)	72 (37%)	12 (34%)	25 (36%)	
Obese (30–39 kg/m^2^)	62 (21%)	53 (27%)	5 (14%)	4 (6%)	
Extreme Obesity (>39 kg/m^2^)	2 (1%)	2 (1%)	0 (0%)	0 (0%)	0.00
**Hypertension**					
No	194 (65%)	124 (64%)	25 (71%)	45 (65%)	
Known diagnosis	64 (21%)	46 (24%)	7 (20%)	11 (16%)	
Measured BP ≥ 140/90 mm/Hg	41 (14%)	25 (13%)	3 (9%)	13 (19%)	0.43
**Diabetes**					
No	241 (81%)	149 (76%)	32 (91%)	60 (87%)	
Known diagnosis	24 (8%)	21 (11%)	0 (0%)	3 (4%)	
Measured HbA1c ≥ 6.5%	34 (11%)	25 (13%)	3 (9%)	6 (9%)	0.09

Abbreviations: BMI = body mass index; BP = blood pressure; HbA1c = hemoglobin A1c.

Table 2 provides a summary of study outcome measures with unadjusted bivariate analysis across the three primary stove types for electrocardiography, echocardiography, ABI, and resting BP. Some measures are continuous, others are binary, indicating the presence or absence of a feature, such as ST depression or elevation. On echocardiography, there was a significant increase in left atrial volume indexed to BSA in participants who were wood stove users, compared to biogas or LPG stove users. The mean left atrial volume for wood stove users was 23 mL/m^2^ compared to 20.8 mL/m^2^ for LPG users There were no statistically significant differencess in left ventricular systolic or diastolic function, right ventricular function, pulmonary pressures, or valvular disease. In general, nearly all participants had normal diastolic function. There was a trend toward larger left ventricular end diastolic volume in wood stove users compared to LPG users. There were no significant differences in ECG between the three groups. Out of the 21 participants that had ST elevations on ECG, there was a trend toward more frequent presence of ST elevations in the group with primary wood stove exposure. However, these ST elevations were consistent with benign early repolarization in 17 participants. In general, participants had normal ABI. The LPG group had a slightly higher mean ABI compared to biogas and wood stove groups. There was no significant difference in systolic or diastolic blood pressure between the three stove use groups.

**Table 2:** Subclinical cardiovascular outcomes with an unadjusted bivariate analysis across 3 primary stove types.

		All Stoves		LPG Stoves		Biogas Stoves		Wood Stoves		All Stoves

	N	Mean or % value	SD	Mean or % value	SD	Mean or % value	SD	Mean or % value	SD	p-value

**Left ventricular systolic function**										
Mean biplane ejection fraction	294	61.6	5.9	61.4	5.9	61.6	6.7	62.2	5.7	0.62
Mean LV mass/BSA (g/m^2^)	298	68.0	16.5	67.9	16.6	68.5	16.2	68.1	16.4	0.98
Mean LV systolic volume/BSA (mL/m^2^)	294	21.1	5.6	21.1	5.5	20.7	6.6	21.5	5.2	0.79
Mean LV diastolic volume/BSA (mL/m^2^)	292	55.2	12.6	54.7	11.7	53.8	14.0	57.4	14.0	0.24
**Left ventricular diastolic function**										
Normal diastolic function, %	284	95%	–	97%	–	91%	–	91%	–	0.14
Mean left atrial volume/BSA (mL/m^2^)	292	21.3	6.0	20.8	6.0	20.4	5.6	23.0	5.7	0.03
**Pulmonary Pressure**										
Mean pulmonary artery systolic pressure (mmHg)	185	25.9	13.4	25.4	11.1	24.1	10.3	28.2	19.2	0.40
Pulmonary hypertension (PASP > 35 mmHg) present, %	299	42%	–	44%	–	37%	–	41%	–	0.70
**Right ventricular function**										
Mean tricuspid annulus S’ velocity (cm/s)	215	11.1	1.5	11.1	1.5	11.0	1.5	11.2	1.5	0.94
Mean RV size (mm)	297	35.0	4.4	35.0	4.3	34.1	4.5	35.2	4.7	0.44
Significant Valvular Disease*, %	299	14%	–	13%	–	9%	–	20%		0.20
**Blood Pressure**										
Mean systolic blood pressure (mmHg)	299	119.4	18.6	119.7	17.5	119.3	23.4	118.6	19.4	0.92
Mean diastolic blood pressure (mmHg)	299	79.6	11.5	79.8	11.3	79.8	14.1	78.9	10.5	0.85
Hypertension (≥140/90 mmHg), %	299	35%	–	36%	–	29%	–	35%	–	0.66
**Electrocardiogram**										
Mean Q-T interval (ms)	299	413.9	25.0	414.0	25.9	408.0	20.3	416.5	24.4	0.26
Q waves present, %	299	9%	–	7%	–	14%	–	10%	–	0.38
ST depression present, %	299	4%	–	4%	–	9%	–	3%	–	0.41
ST elevation present, %	299	7%	–	5%	–	9%	–	13%	–	0.07
T-wave abnormal, %	299	14%	–	15%	–	9%	–	14%	–	0.53
ST/T-wave abnormality**, %	299	23%	–	21%	–	26%	–	28%	–	0.51
**Mean ankle-brachial index**	299	1.1	0.1	1.2	0.1	1.1	0.1	1.1	0.1	0.05

Abbreviations: LV = left ventricle; BSA = body surface area; RV = right ventricle; PASP = pulmonary artery systolic pressure. * Significant valvular disease: any degree of stenosis of any cardiac valve, any aortic regurgitation, or greater than mild tricuspid or mitral regurgitation. ** ST/T-wave abnormalities: composite of ST-segment depressions, elevations or T-wave abnormalities.

Table 3 shows the linear multivariable regression results of the exposure variables on different outcomes. Notably, after adjustment by covariates, left ventricular end diastolic volumes and left atrial volumes were significantly larger in wood stove users compared to LPG stove users. ABI, systemic blood pressure, left ventricular mass, pulmonary artery systolic pressures, and right ventricular function were not significantly different amongst the participants. Table 4 presents the results of the logistic multivariable regressions of ECG outcomes, showing no significant difference in ECG characteristics between the stove use groups.

**Table 3:** Multivariate linear regression results for the use of biogas and wood stoves vs. liquified petroleum gas (LPG) stoves for a series of different cardiovascular outcomes.

	Biogas Stove β (95% CI)^a^	P-value	Wood Stove β (95% CI)^a^	P-value

Left ventricular mass indexed to BSA (g/m^2^)	0.90 (–5.52, 7.32)	0.78	–0.96 (–7.06, 5.13)	0.76
Left ventricular end diastolic volume indexed to BSA (mL/m^2^)	2.34 (–2.60, 7.29)	0.35	7.97 (3.11, 12.83)	0.001
Left ventricular end systolic volume, indexed to BSA (mL/m^2^)	0.54 (–1.87, 2.95)	0.66	1.90 (–0.12, 3.93)	0.07
Left ventricular ejection fraction, %	0.84 (–1.85, 3.53)	0.54	1.74 (–0.65, 4.13)	0.15
Pulmonary artery systolic pressure (mmHg)	–7.62 (–21.69, 6.45)	0.29	–0.45 (–14.23, 13.34)	0.95
Right ventricular basal diameter (mm)	–1.66 (–4.10, 0.78)	0.18	–0.37 (–3.26, 2.53)	0.80
Right ventricular mean tricuspid annulus S’ velocity (cm/s)	7.52 (–7.83, 22.88)	0.34	4.31 (–10.33, 18.95)	0.56
Left atrial volume indexed to body surface area (mL/m^2^)	0.44 (–1.64, 2.51)	0.68	3.15 (1.22, 5.09)	0.001
ABI	–0.02 (–0.05, 0.00)	0.10	–0.01 (–0.04, 0.02)	0.43
QT interval (ms)	–4.91 (–13.07, 3.26)	0.24	–0.52 (–9.30, 8.27)	0.91
Systolic BP	2.19 (–6.15, 10.52)	0.61	–1.52 (–8.56, 5.52)	0.67
Diastolic BP	0.75 (–4.45, 5.95)	0.78	–1.60 (–6.19, 2.98)	0.49

Abbreviations: BSA = body surface area; ABI = ankle brachial index; BP = blood pressure. ^a^ Adjusted for age, BMI or BSA, education, diabetes, smoking history, urban/rural residence, presence of house heating, and fuel use for lighting.

**Table 4:** Multivariate unconditional logistic regression analyses evaluating associations of primary stove type (wood or biogas vs. liquid petroleum gas) with changes in ECG outcomes.

ECG characteristic (Minnesota code)	Biogas Stove Odds ratio (95% CI)^a^	P-value	Wood Stove Odds ratio (95% CI)^a^	P-value

Q waves present (1-1-1, 1-1-2, 1-1-3, 1-1-4)	3.26 (0.83, 12.79)	0.09	3.11 (0.78, 12.45)	0.11
ST depression present (4-1-1, 4-1-2, 4-2)	3.97 (0.80, 19.82)	0.09	1.01 (0.19, 5.26)	0.99
ST elevation present (9-2)	1.95 (0.38, 9.97)	0.42	1.46 (0.37, 5.70)	0.59
T-wave abnormality present (5-1, 5-2, 5-3)	0.40 (0.10, 1.52)	0.18	0.43 (0.15, 1.26)	0.12
ST/T wave abnormality present (4-1-1, 4-1-2, 4-2, 9-2, 5-1, 5-2, 5-3)	1.08 (0.40, 2.89)	0.87	0.72 (0.31, 1.67)	0.45

^a^ Adjusted for age, BMI, education, diabetes, smoking history, urban/rural residence, presence of house heating, and fuel use for lighting.

Supplemental Tables 2 and 3 show additional analyses adjusting for blood pressure as a covariate, with unchanged results, suggesting that blood pressure does not mediate our observed effects.

## Discussion

This cross-sectional study, which evaluated associations between primary cooking fuel type and measures of cardiovascular structure and function, suggests associations of both increased left atrial volume and increased left ventricular end diastolic volume with use of wood-burning primary cookstoves, compared to using primary LPG stoves. Notably, we did not find an association between biomass fuel exposure and hypertension, as has been previously shown [3,5,22]. Additionally, we did not find significant differences in cardiac function, pulmonary pressures, ABI, or ECG characteristics based on biomass exposure. No differences between biogas and LPG users were found, although the number of biogas users in the study was small.

In general, increased left atrial volumes and left ventricular volumes may be due to pressure or volume overload associated with many cardiomyopathies, heart failure, diastolic dysfunction, tachyarrhythmias, or valvular disease. The vast majority of participants in our study had normal diastolic function when classified according to the 2016 ASE guidelines, excluding diastolic dysfunction as the likely etiology for the atrial enlargement seen in this study [24]. Other commonly recognized causes of increased left atrial and left ventricular volume are mitral valvular disease and tachyarrhythmias. However, only one participant in our study had significant mitral valve disease (classified as any amount of mitral stenosis or greater than mild regurgitation), and none had ECG evidence of tachyarrhythmias. In the absence of continuous telemetry monitoring, we cannot exclude the possibility of an unrecognized arrhythmic contribution to our observations. Despite a statistically significant difference in left atrial volumes, the mean left atrial volume for primary wood stove users remained within the normal range, as defined by the ASE guidelines, at 23 mL/m^2^ compared to 20.8 mL/m^2^ for LPG users [19]. Similarly, the mean left ventricular end diastolic volume indexed to BSA for wood stove users was within the normal range at 57.4 mL/m^2^, and 54.7 mL/m^2^ for LPG users. These normative values were established in European and American populations, and it is not known if they are appropriate for this population of Nepali women. The observed trend of increased left ventricular and atrial volumes may be a result of direct adverse cardiac remodeling and fibrosis stimulated by HAP. Studies in rats have previously documented the role of fine particulate matter (PM_2.5_) in stimulating cardiac fibrosis [14,34].

The possibility also exists that the associations we found could have been a function of the multiple comparisons we examined in this analysis. Hence, we consider our provocative findings of atrial and ventricular diastolic volume to be observations that will require replication in future studies.

We anticipated finding biomass smoke effects on BP and on cardiac end-organ measures most typical for sustained elevated BP, such as increased left ventricular mass. We did not find these associations in this study. The possible presence of masked hypertension may account for why we did not observe BP elevation. Prior studies evaluating masked hypertension have shown an 8–20% prevalence in the general population and up to 50% prevalence in treated hypertensive patients, although the Nepali population has not been specifically studied [9,27]. It is also possible that prior studies have found an association with hypertension and HAP because such studies relied on field evaluations [5,14,16]. Transient blood pressure effects similar to “white coat” hypertension may have been attenuated in our study given that study participants had to travel to Pokhara for evaluation. However, with longstanding hypertension, we would expect to find changes in the myocardium with increased left ventricular mass and evidence of diastolic dysfunction, but we did not detect these changes in this study.

Overall, there is a lack of data from low- and middle-income countries on subclinical and clinical risk of HAP and cardiovascular disease. Results from this study add to the existing literature by describing the prevalence of cardiovascular risk factors in this particular population and by evaluating potential cardiac effects of HAP. A prior study evaluating echocardiography results in daily biomass fuel users compared with non-users found an increase in left ventricular and left atrial size. However, when adjusted for education and wealth, there was no difference between the two groups [28]. Another smaller scale study with 44 participants in Kenya found an association between carbon monoxide and fine particular matter exposure and decreased left ventricular ejection fraction [1].

The present study is one of the largest and most comprehensive evaluations of the potential adverse subclinical cardiac effects of HAP. The major limitation of the project is the cross-sectional design, which precluded identification of changes that developed over time with exposure. In addition, we had anticipated a closer balance between the size of the groups for the three primary cookstove types (wood, LPG, and biogas). The imbalance is attributable to monsoon rains coinciding with the start of the study. Given difficulties with transportation during the monsoon, more participants were recruited from the urban areas near the field office in Pokhara. Urban families are much more likely to use LPG than rural families, resulting in two different populations with different risk factors and levels of risk for cardiovascular disease. We adjusted for urban/rural residence location and education to account for these differences. Because of the restricted sample size, exposure assessment in this analysis is limited to the primary stove type used by participants, rather than a broader range of potential airborne exposures (e.g., from heating, incense burning, mosquito coils, and oil lamps) that participants were potentially exposed to.

The major strengths of this study include that it had a very high participation rate (98%) and is the first study to carry out a comprehensive suite of hospital-based cardiology tests in a relatively large sample of women using a variety of cook stove types. Prior studies with comparable methodology have had sample sizes ranging from 44 to 389 participants [1,5,26,30]. Given the size and comprehensive nature of evaluation, this study allowed us to describe the prevalence of cardiovascular risk factors in Nepalese women and to evaluate the association of subclinical cardiovascular changes with various stove types. Additionally, we separately categorized LPG and biogas stoves, rather than just as “gas”, as has been the case in most previous studies. LPG is propane and butane and burns with a higher temperature than biogas, which is mostly methane and carbon dioxide.

In conclusion, we found an association between increased left atrial volume, and increased left ventricular end diastolic volumes with wood stove exposure in women in Nepal. These results, if separately confirmed, may provide insight into potential subclinical cardiovascular disease related to HAP.

## Additional Files

The additional files for this article can be found as follows:

10.5334/gh.405.s1 Supplemental Table 1. Baseline characteristics by primary stove type for 299 Nepali women, Kaski District, Nepal.

10.5334/gh.405.s2 Supplemental Table 2. Multivariate linear regression including adjustment for blood pressure for the use of biogas and wood stoves vs. liquified petroleum gas (LPG) stoves for a series of different cardiovascular outcomes.

10.5334/gh.405.s3 Supplemental Table 3. Multivariate unconditional logistic regression analyses including adjustment for blood pressure and evaluating associations of primary stove type (wood or biogas vs. liquid petroleum gas) with changes in ECG outcomes.
