# Supplementary material for: Biomass Fuel Use and Cardiac Function in Nepali Women
Source: Glob Heart. 2020 Feb 7;15(1):11. doi: 10.5334/gh.405 (PMC7218789; doi:10.5334/gh.405)
Supplement: Supplemental Table 1. — Baseline characteristics by primary stove type for 299 Nepali women, Kaski District, Nepal. [file gh-15-1-405-s1.pdf]

Supplemental Table 1. Baseline characteristics by primary stove type for 299 Nepali women, Kaski District, Nepal.

		All Subjects - Current House			
	All Stoves (N=299)	LPG (N=195)	Biogas (N=35)	Wood (N=69)	X^2 value across stove types
	N (%)	N (%)	N (%)	N (%)	P value
**Secondary stoves (different to primary stove)**					
LPG	50 (17%)	0 (0%)	26 (74%)	24 (35%)	0.00
Biogas	27 (9%)	22 (11%)	0 (0%)	5 (7%)	0.08
Wood	42 (14%)	29 (15%)	13 (37%)	0 (0%)	0.00
Kerosene	1 (0%)	1 (1%)	0 (0%)		0.77
Electric	9 (3%)	9 (5%)	0 (0%)	0 (0%)	0.08
Rice Cooker	162 (54%)	138 (71%)	12 (34%)	12 (17%)	0.00
Coal	33 (11%)	5 (3%)	7 (20%)	21 (30%)	0.00
**Worked overseas**	1 (0%)	1 (1%)	0 (0%)	0 (0%)	0.77
**Religion**					
Hindu	269 (90%)	174 (89%)	35 (100%)	60 (87%)	
Buddhist	20 (7%)	13 (7%)	0 (0%)	7 (10%)	
Christian	5 (2%)	5 (3%)	0 (0%)	0 (0%)	
Other	5 (2%)	3 (2%)	0 (0%)	2 (3%)	0.24
**Caste**					
Dalit	33 (11%)	22 (11%)	2 (6%)	9 (13%)	
Disad. Janajatis	45 (15%)	32 (16%)	4 (11%)	9 (13%)	

Religious Minority	2 (1%)	2 (1%)	0 (0%)	0 (0%)	
Relative Adv. Janajat	47 (16%)	34 (18%)	2 (6%)	11 (16%)	
Upper Caste	171 (57%)	104 (54%)	27 (77%)	40 (58%)	0.39
**Family owns land**	289 (97%)	189 (97%)	34 (97%)	66 (96%)	0.87
**Kitchen location**					
Inside house	237 (80%)	150 (77%)	28 (80%)	59 (87%)	
Attached to house	38 (13%)	28 (14%)	4 (11%)	6 (9%)	
Unattached to house	20 (7%)	15 (8%)	2 (6%)	3 (4%)	
Outdoor	2 (1%)	1 (1%)	1 (3%)	0 (0%)	0.45
**House ownership**					
Own	279 (93%)	178 (91%)	35 (100%)	66 (96%)	
Rent	20 (7%)	17 (9%)	0 (0%)	3 (4%)	0.11
**Household crowding**					
<= 2 people per room	290 (97%)	189 (97%)	35 (100%)	66 (96%)	
> 2 people per room	9 (3%)	6 (3%)	0 (0%)	3 (4%)	0.47
**Household has transportation**	169 (57%)	131 (67%)	21 (60%)	17 (25%)	0.00
**Lighting when electricity unavailable**					
Kerosene Lamp	7 (2%)	0 (0%)	0 (0%)	7 (10%)	0.00
Candles	24 (8%)	15 (8%)	2 (6%)	7 (10%)	0.70
Solar Lamp	160 (54%)	109 (56%)	25 (71%)	26 (38%)	0.00
Battery Lights	105 (35%)	60 (31%)	9 (26%)	36 (52%)	0.00

Oil Lamp	10 (3%)	2 (1%)	0 (0%)	8 (12%)	0.00
Generator	2 (1%)	2 (1%)	0 (0%)	0 (0%)	0.59
**Burn mosquito coils**	49 (16%)	34 (17%)	6 (17%)	9 (13%)	0.69
**Burn incense**	199 (67%)	123 (63%)	27 (77%)	49 (71%)	0.18
**Heat Home**	47 (16%)	23 (12%)	2 (6%)	22 (32%)	0.00
**Miss/no food in 6 months**	1 (0%)	1 (1%)	0 (0%)	0 (0%)	0.77
**Time spent in kitchen during cooking:**					
All of the time	32 (11%)	18 (9%)	1 (3%)	13 (19%)	
Most of the time	58 (19%)	44 (23%)	1 (3%)	13 (19%)	
Some of the time	185 (62%)	117 (60%)	31 (89%)	37 (54%)	
Little of the time	17 (6%)	10 (5%)	2 (6%)	5 (7%)	
No time	6 (2%)	6 (3%)	0 (0%)	0 (0%)	
Outside Kitchen	1 (0%)	0 (0%)	0 (0%)	1 (1%)	0.01
**Lived in current house (years)**					
0-10	73 (24%)	63 (32%)	5 (14%)	5 (7%)	
10-20	85 (28%)	60 (31%)	10 (29%)	15 (22%)	
20-30	83 (28%)	52 (27%)	11 (31%)	20 (29%)	
30-70	58 (19%)	20 (10%)	9 (26%)	29 (42%)	0.00

Abbreviations: LPG= liquid petroleum gas
