# Supplementary material for: Biomass Fuel Use and Cardiac Function in Nepali Women
Source: Glob Heart. 2020 Feb 7;15(1):11. doi: 10.5334/gh.405 (PMC7218789; doi:10.5334/gh.405)
Supplement: Supplemental Table 2. — Multivariate linear regression including adjustment for blood pressure for the use of biogas and wood stoves vs. liquified petroleum gas (LPG) stoves for a series of different cardiovascular outcomes. [file gh-15-1-405-s2.pdf]

Supplemental Table 2. Multivariate linear regression including adjustment for blood pressure for the use of biogas and wood stoves vs. liquified petroleum gas (LPG) stoves for a series of different cardiovascular outcomes.[a]

	**Biogas Stove β (95% CI)[b]**	**P- value**	**Wood Stove β (95% CI)[b]**	**P- value**
Left ventricular mass indexed to BSA (g/m^2)	0.34 (-5.86, 6.55)	0.91	-0.61 (-6.38, 5.15)	0.83
Left ventricular end diastolic volume indexed to BSA (mL/m^2)	2.07 (-2.80, 6.95)	0.40	8.17 (3.43, 12.92)	0.00
Left ventricular end systolic volume, indexed to BSA (mL/m^2)	0.44 (-1.96, 2.84)	0.72	1.96 (-0.02, 3.95)	0.052
Left ventricular ejection fraction, %	0.81 (-1.88, 3.50)	0.55	1.76 (-0.66, 4.17)	0.15
Pulmonary artery systolic pressure (mmHg)	-7.52 (-21.78, 6.75)	0.30	0.41 (-13.39, 14.21)	0.95
Right ventricular basal diameter (mm)	-1.67 (-4.13, 0.80)	0.19	-0.38 (-3.34, 2.57)	0.80
Right ventricular mean tricuspid annulus S' velocity (cm/s)	7.20 (-8.26, 22.65)	0.36	4.42 (-10.14, 18.99)	0.55
Left atrial volume indexed to body surface area (mL/m^2)	0.36 (-1.77, 2.49)	0.74	3.08 (1.17, 4.99)	0.00
ABI	-0.02 (-0.05, 0.00)	0.10	-0.01 (-0.04, 0.02)	0.43
QT interval (ms)	-5.00 (-13.20, 3.20)	0.23	-0.08 (-8.96, 8.79)	0.99
Systolic BP	2.19 (-6.15, 10.52)	0.61	-1.52 (-8.56, 5.52)	0.67
Diastolic BP	0.75 (-4.45, 5.95)	0.78	-1.60 (-6.19, 2.98)	0.49

Abbreviations: BSA= body surface area; ABI= ankle brachial index; BP = blood pressure

a. Supplemental analysis to Table 3 which does not include adjustment for pulse pressure and diastolic pressure.

Adjusted for age, BMI or BSA, pulse pressure and diastolic blood pressure (used for all outcomes except blood pressure), education, diabetes, smoking history, urban/rural residence, presence of house heating, and fuel use for lighting.
