# Supplementary material for: Biomass Fuel Use and Cardiac Function in Nepali Women
Source: Glob Heart. 2020 Feb 7;15(1):11. doi: 10.5334/gh.405 (PMC7218789; doi:10.5334/gh.405)
Supplement: Supplemental Table 3. — Multivariate unconditional logistic regression analyses including adjustment for blood pressure and evaluating associations of primary stove type (wood or biogas vs. liquid petroleum gas) with changes in ECG outcomes. [file gh-15-1-405-s3.pdf]

Supplemental Table 3. Multivariate unconditional logistic regression analyses including adjustment for blood pressure and evaluating associations of primary stove type (wood or biogas vs. liquid petroleum gas) with changes in ECG outcomes.

**ECG characteristic (Minnesota code)**	**Biogas Stove Odds ratio (95% CI)[b]**	**P- value**	**Wood Stove Odds ratio (95% CI)[b]**	**P- value**
Q waves present (1-1-1, 1-1-2, 1-1-3, 1-1-4)	3.26 (0.81, 13.13)	0.09	3.14 (0.79, 12.48)	0.10
ST depression present (4-1-1, 4-1-2, 4-2)	4.31 (0.75, 24.69)	0.10	0.91 (0.15, 5.51)	0.92
ST elevation present (9-2)	1.78 (0.34, 9.46)	0.50	1.34 (0.35, 5.16)	0.67
T-wave abnormality present (5-1, 5-2, 5-3)	0.39 (0.10, 1.46)	0.16	0.40 (0.14, 1.15)	0.09
ST/T wave abnormality present (4-1-1, 4-1-2, 4-2, 9-2, 5-1, 5-2, 5-3)	1.04 (0.40, 2.72)	0.94	0.68 (0.29, 1.58)	0.37

a. Supplemental analyses to Table 4 which does not include adjustment for pulse pressure and diastolic pressure.

Adjusted for age, BMI, education, diabetes, smoking history, urban/rural residence, presence of house heating, pulse pressure, diastolic blood pressure, and fuel use for lighting.
